# Caring for the Guardians—Exploring Needed Directions and Best Practices for Police Resilience Practice and Research

**DOI:** 10.3389/fpsyg.2020.01874

**Published:** 2020-08-21

**Authors:** Olivia Carlson-Johnson, Heath Grant, Cathryn F. Lavery

**Affiliations:** ^1^Institute for Intergovernmental Research, Belleville, IL, United States; ^2^John Jay College of Criminal Justice, New York, NY, United States; ^3^Pace University, Pleasantville, NY, United States

**Keywords:** compassion fatigue, resilience, Police suicide, stress, Trauma

## Introduction

Occupational groups that experience heightened levels of stress and trauma, such as law enforcement can be at increased risk for long-term health care issues, mental health concerns, psychological disorders, and behavioral issues (Berg et al., 2003; Waters and Ussery, 2007; Griffin et al., 2010; IACP, 2014; Korre et al., 2014; Tucker, 2015). Law enforcement officers are often exposed to death, violence, and other forms of human misery. However, it is often not a single incident or event that can lead to catastrophic issues.

Often, it is the cumulative effects of such exposures, coupled with the lack of appropriate professional assistance that can lead to negative outcomes (Berg et al., 2003; Griffin et al., 2010; IACP, 2014).

Research notes that law enforcement as a profession is also at increased risk for completed suicide (Kelly and Martin, 2006; Violanti, 2007; The Badge of Life, 2008–2012; Violanti et al., 2013, 2016). In fact, officers are at greater risk of dying by suicide, then by being killed in the commission of their duties (Heyman et al., 2018). Nevertheless, it should be noted that suicide among this occupational group is not a new phenomenon within the general population, where 13 out of 100,000 die by suicide. However, for police it is now at a rate of 17 per 100,000 (Heyman et al., 2018). In fact, suicide has been a leading killer of law enforcement officers for years (Kelly and Martin, 2006; Violanti, 2007). Indeed, the recent President's Task Force on twenty-first Century Policing (2015) already anchored concern for officer wellness as one of its six pillars.

Unfortunately, 2019 also brought a surge in the numbers of police suicides in the United States, provoking heightened media attention that lead administrators and experts to even consider the possibility of contagion within the larger mental health concerns facing police. Suicide clusters within single agencies, such as the New York City Police Department (NYPD) contributed to an almost frenzied response by administrators and experts to try and find a solution. Regrettably, to date, there has been insufficient research available on the causes of police suicide and evidence- based responses been developments within the field of positive psychology that can be turned to search for practical and meaningful applications for the law enforcement field. Additionally, the military has successfully implemented various approaches to resiliency training that should be transferable to law enforcement environments. However, although the military culture and organization has obvious similarities to policing, such interventions still need to be rigorously tested with law enforcement (Chopko and Schwartz, 2013).

Importantly, police officers themselves have long recognized the need for some intervention and help related to wellness. Heyman et al. (2018) found that over 35% of police characterize themselves experiencing a form of post- traumatic stress disorder (PTSD) compared to 6.8% of the general population. Over 12% officers indicated that they believe they experience depression, vs. 7% of the mass population. This is concerning but most likely under-reported figures due to the nature of the field (Heyman et al., 2018; LET Staff, 2018). The organizational culture of most law enforcement agencies makes many officers afraid to report their psychological stress or issues out of fear of being relegated to administrative work as a response.

The public is aware of the inherent dangers of the field of law enforcement. In addition to imminent physical injury or death, police officers are the gate keepers of public safety—they are on the front lines of our communities and must deal with a myriad of stressful and continuous issues. Law enforcement officers are the first to speak with victims, to respond to crime scenes, and in pursuing suspects. They are constantly exposed to and witness disturbing circumstances ranging from murder, sexual assault, intimate partner violence and child abuse. On average, law enforcement witness approximately 188 critical incidents during their tenure (Heyman et al., 2018). Exposure to such events may lead to significant multiple psychological, mental and emotional traumas. Most officers have been conditioned thorough their subculture to remain quiet of their feelings of mental and emotional stress. This has been proven to create on-going symptoms of emotional withdrawal, burnout, and excessive fatigue, neglect of self-care which in turn, has an enormous impact on their intimate, social, work and community relationships.

It should also be stressed that officers are more likely to receive assistance immediately following critical incidents, but that the long-term effects of both primary and secondary trauma (as in the case of compassion fatigue discussed below) can emerge either suddenly or after a significant period of time.

## Compassion Fatigue

Compassion fatigue was first introduced in the medical field in 1992 by Carla Joinson, RN. In her description, she described a reaction similar to burnout in which a caregiver experiences “a loss of the ability to nurture” (Joinson, 1992, p. 119). Compassion fatigue interacts with a combination of stressors ranging from long work hours, heavy caseloads, responding to trauma and the care giver feeling detached, inefficient, over tired, angry and dissatisfied with their job (Joinson, 1992, p. 118–120). Figley, initially examined “secondary traumatic stress (STS) to describe compassion fatigue (Figley, 1999). Figley suggested that” perhaps PTSD should stand for primary traumatic stress disorder, rather than posttraumatic stress disorder because every stress reaction is “post” by definition (Figley, 1999). In addition, he wrote that caregivers experience pain from their exposure to someone's trauma (immediate situation). “This situation—call it compassion fatigue, compassion stress or secondary traumatic stress—is the natural, predictable, treatable and preventable unwanted consequence of working with suffering people” (Brown et al., 1999; Figley, 1999). When the practitioner is overstressed, compassion is weakened. The practitioner can display similar symptoms triggered by those they are helping. Individual as well as external stressor can lead to job dissatisfaction, negatively impact their home environments and ultimately became an “occupational hazard” (Mathieu, 2007). Figley (1999), described compassion fatigue as “the cost of caring” for those affected by their work and continually seeing others in pain and experiencing trauma. Those in fields with either direct exposure to traumatic effects (police, emergency hospital workers, nurses, etc.) or even secondary exposure (listening to victims' experiences, child protection issues, etc.).

Compassion fatigue has been examined in the areas of emergency rooms, veterinarians, nurses, etc. but has only been recently explored with law enforcement officers (Papazoglou and Andersen, 2014, p. 5; Grant et al., 2019, p. 5–7). To complicate matters with police officers, it is likely that the increased levels of poor morale amongst police as a result of negative interactions with the community in recent years could also contribute to the burnout that is a central component of compassion fatigue.

A study conducted at the National Institute of Ethics (2012) found that there exist multiple sources of frustration and anger experienced by law enforcement officers, with the common denominator being administration. Examples of organizational problems, unpredictable discipline, politics and favoritism all contribute to on-going feelings of anxiousness that are compounded by trauma they experience on duty (Cruickshank, 2012). These organizational factors are risk factors for stress and fatigue that must be addressed as part of any intervention targeting sustainable improvements in the overall wellness of their officers (Blumberg et al., 2020). As will be discussed below, successful interventions targeting individual officers will have less impact if they do not also address the complexity of stressors (organizational community, etc.) contributing to the deteriorating wellbeing of first responders (NBC-LA Los Angeles Southern California, 2019; Blumberg et al., 2020).

## Other Essential Contributing Factors

Research suggests that when compared to the general population, law enforcement professionals are generally more resilient (Galatzer-Levy et al., 2011; Papazoglou et al., 2017). However, certain occupational components actually contribute to the continued attrition of officer health and wellness. The ambiguous nature of police work requires officers to fill many roles and act in innumerable roles. The ambiguity of the police role, the extremes of policing, and the constant and continuous exposure to stress, trauma, and life-threatening incidents place officers at risk for mental health issues, stress-induced illnesses, and even completed suicide (Paton et al., 2008) Police work can be extremely rewarding. However, the flip side of this service-oriented occupation includes increased concentrations of stress, trauma, and adversity when compared to the general public (Johnson et al., 2019).

Numerous factors contribute to the stress and trauma experienced by police officers. Officers often find it difficult to just shut off when they go off-duty. Many will remain vigilant and even hyper-vigilant off-duty until their next shift. This constant barrage of stress-inducing behavior is counter-productive and unhealthy.

In addition to on-duty internal stressors, officers also face many common everyday stressors (financial concerns, relationship issues, health issues, etc.). Unfortunately, these same men and women who are deemed problem-solvers and are expected (whether societally or personally) to keep their issues private, resulting in many to suffer silently. Officers may revert to maladaptive coping to deal with the stress (e.g., excessive use of alcohol, drug use, gambling, sex addictions, shopping, etc.). These maladaptive coping mechanisms will also create additional issues and stress, becoming a vicious cycle if not broken.

These internal stressors are, of course, important and the most logical for many agencies to immediately identify and understand. However, organizational stressors = departmental policies and procedures, sifting through the red tape and bureaucracy that plagues many agencies—can be equally dangerous. This is why comprehensive, multi-level organizational responses that go beyond typic academy and in-service trainings are argued for here so strongly.

As alluded to above, external factors include the more obvious inherent dangers of police work. Approximately 160 officers are killed each year in the line of duty (Officer Down Memorial Page, 2009-2018). In addition to those who pay the ultimate price, thousands more are injured and assaulted, with a percentage of these officers never to return to duty (National Law Enforcement Officers Memorial Fund, 2019).

A lack of positive interactions with the community can also play an important role in laying a toxic foundation for overall officer wellness. Research has shown that most officer come to the profession with a strong service-orientation to make a difference. When some or most of their interactions with the community is negative, this disconnect can be a major factor in the onset of compassion fatigue. Additionally, officers most often see only the negative side of the incidents they are involved in. Often, they are not informed of the later positive outcomes of situations they have responded to (family reconciliation, recovery of victims, etc.). Combined, these external factors can diminish the sense of control, optimism, and hopefulness that research has shown to provide essential recovery capital for police officer overall wellness and resilience (Grant et al., 2019).

## Evidence-Based Interventions to Promote Police Officer Resilience

Rather than a negative perspective to understanding officer wellness, it is important to recognize what makes most officers able to perform effectively through (or bounce back after) the many stressful and traumatic incidents they face throughout their careers. Although police officers are at a general risk for the stress that can impact both their physical and mental health, the field of positive psychology suggests that interventions need to target building the positive skills known to buffer against the risk factors influencing the problem.

Recent research offers an important possible avenue for such interventions. If officers are losing their connection to the positive, helping aspects of the job that they entered the profession for in the first place, multilevel interventions should find a way to reestablish this. Compassion satisfaction refers to this satisfaction that people get from the helping aspects of professions such as policing, nursing etc. Grant et al. (2019) demonstrated that compassion satisfaction was significantly lower in their sample of police officers as compared to the general population.

Even in their sample compassion satisfaction was highly correlated with compassion fatigue. As compassion satisfaction increases, compassion fatigue decreases, suggesting that interventions seeking to address officer resilience need to target this essential protective factor.

Existing interventions in policing targeting compassion satisfaction related skills and attitudes fall under the modern servant leadership movement (Badger, 2019). Profiled in the *Law Enforcement Bulletin*, Efforts to promote servant leaders in policing seek to instill “the natural feeling that one wants to serve, to serve *first.”* Importantly, this approach represents the multilevel intervention that is required for sustainable impacts (Blumberg et al., 2020). This is because servant leadership builds the organizational environment for building officer resilience by offering officers meaningful ways to improve “mental, physical, spiritual, and social fitness.” Offering officers, the requisite autonomy and control fosters the development of compassion satisfaction that makes officers more optimistic and positive about their job, even as they are faced with the external factors that are a real part of their daily work. As such, servant leadership creates collaborative environments that allows the voice of each officer to be heard.

Of course, some individual level interventions are also important. Some skill-based programs teaching officers how to regain psychological balance after challenging situations have been demonstrated to be effective (McCraty and Atkinson, 2012). Police officers participating in the Stress Resilience Training Program in San Diego reported improvements in overall stress reductions in work, personal, and family situations. This program used an I-Pad system to develop these skills over time.

The HEROES Project represents a very promising practice to provide officers the cluster of skills needed for resilience building. Importantly, this is a 8 week online training program that focusing on skill-building in areas known to be core to resilience, such as mindfulness and spirituality. Created by Command Post President, Renee Thornton in 2010, exercises are scaffolded to build over time, an educational resource platform dedicated to officer wellness online makes it scalable and more practical for most law enforcement agencies. Preliminary research indicates that HEROES significantly reduced stress, depression, anxiety, and trauma for all participants (Thornton, 2019). The research on HEROEs (Thornton et al., 2019) is growing as several cohorts of police officers and veterans have participated in the program over the last several years. Additional replication evaluation studies are currently underway.

The multilevel nature of risk and protective factors suggests the need for multilevel programs. The general positive psychology and resilience shows that the more risk factors targeted by programs exponentially decreases the likelihood of the related problem behavior. There is no reason to suggest that this is not true for policing. Indeed, it is likely to be even more true given the unique nature of the job.

Finally, on the individual level, there has been a large degree of interest in emotional intelligence interventions or empathy training programs as well, with some evidence-base suggesting that emotional intelligence is significantly associated with overall mental health (Schutte et al., 2013) that could be related to burnout, trauma, and even suicide at the extreme.

## Recommendations and Moving Forward

Los Angeles Police Department, once dealing with 37 suicides from 2008 to 2016, has been an example of success for attempting to assist in lowering the rate of depression and suicide within its agency. Between 2017- October of 2019, there have been no suicides. LAPD instituted a new peer support initiative. Although participant numbers are low (notably due to subculture and stigma associated with mental health issues) and no documented evaluation has been done to date, the reflection in the numbers are promising. LAPD's initiative has led to other police agencies taking similar steps in a prevention direction and shedding more light and awareness on police stress and the impact on officers their respective agencies. Lokkesmoe (2018) has discussed law enforcement agencies that have taken certain steps to help in preventing depression, burnout, and suicides since earlier programs like LAPD's have emerged. These include: stronger dissemination of information for new hires about on the job stress and its impact; opportunities and care available for current employees, and the availability of resources for senior level administrators and all officers. Another recommendation has been to develop more officer self-awareness programming and resources on mental health as well as incorporating on-going mental health training and awareness in academies and all departmental agencies. It is believed that this would not only help officers' from feeling possible stigmatization but also bring further light and awareness to mental health issues existing in their communities (Health Matters, 2019).

Even with an emerging program evaluation record, the approaches discussed here need additional research, and can only be considered “promising practices” at this stage. The lack of empirical study of the interventions to date is a symptom of the same lack of empirical study of the problems themselves (e.g., compassion satisfaction, officer suicide, officer burnout and trauma etc.). A central purpose of this article is to encourage such academic study of both problem and its most effective responses to date.

Such empirical attention to police officer mental health is very important and timely, in light of the growing concern around officer suicide. One group bringing attention to officer suicide in meaningful way with a never before project is 1 Is Too Many®. This group tracks suicide and murder-suicide deaths of police and correctional officers nationwide to include active, former, and retired members. This large-scale study will address over 50 data points in reported cases of officer suicide and murder-suicide from 2017 to 2019. Based on the data collected, a partial psychological autopsy will be developed to provide insight into the life and death of the decedent, contributing and protective factors, possible motives, suicidal intent, behavior modifications, personality changes, etc. Once complete, this research will be used to assist law enforcement agencies in developing and implementing policies and procedures for reducing suicide risk through early intervention programs and proactive risk assessment. Guidelines will also be established for police families witnessing warning signs or problematic behavior. Lastly, this study aims to provide a better understanding suicide among these first responder populations in a way that has never been done before. The authors of this paper are also planning a large-scale expansion replication study to further understand the role of compassion fatigue in overall officer wellness.

## Author Contributions

OC-J focused on research on police suicide and literature review. HG focused on resiliency and prior research. CL focused on history of compassion fatigue and literature review. All authors reviewed preliminary edits before submission.

## Conflict of Interest

The authors declare that the research was conducted in the absence of any commercial or financial relationships that could be construed as a potential conflict of interest.
